# A novel role of kukoamine B: Inhibition of the inflammatory response in the livers of lipopolysaccharide-induced septic mice via its unique property of combining with lipopolysaccharide

**DOI:** 10.3892/etm.2015.2188

**Published:** 2015-01-19

**Authors:** WEI-TING QIN, XU WANG, WEI-CHANG SHEN, BING-WEI SUN

**Affiliations:** Department of Burns and Plastic Surgery, Affiliated Hospital, Jiangsu University, Zhenjiang, Jiangsu 212001, P.R. China

**Keywords:** kukoamine B, lipopolysaccharide, liver, inflammatory response, nuclear factor κ-light-chain-enhancer of activated B cells

## Abstract

Kukoamine B (KB), derived from the traditional Chinese herb cortex Lycii, exerts anti-inflammatory effects due to its potent affinity with lipopolysaccharide (LPS) and CpG DNA; however, little is known regarding whether the *in vivo* administration of KB can effectively inhibit inflammation in septic mice. The present study thus aimed to investigate the inhibitory effects of KB on the inflammatory response in the livers of LPS-induced septic mice. KB treatment in the LPS-induced septic mice significantly decreased the plasma level of LPS. In addition, KB protected against liver injury, as confirmed by improved histology and decreased aminotransferase levels in the serum. Further experiments revealed that KB attenuated liver myeloperoxidase activity and reduced the expression of vascular cell adhesion molecule-1 and intercellular adhesion molecule-1. These effects were accompanied by decreases in the levels of tumor necrosis factor α and interleukin-1β in the liver tissue. In parallel, the activity of nuclear factor-κ-gene binding (NF-κB) in the livers of LPS-induced septic mice was markedly inhibited with KB treatment. In combination, these results demonstrate that KB inhibits inflammation in septic mice by reducing the concentrations of plasma LPS, decreasing leukocyte sequestration and interfering with NF-κB activation, and, therefore, suppressing the pro-adhesive phenotype of endothelial cells.

## Introduction

Sepsis is a combination of clinical manifestations of systemic inflammation specifically associated with an infectious insult (1). The condition is the most frequent cause of mortality in the majority of intensive care units and is responsible for >250,000 mortalities in the United States annually (2). Despite advances in basic and clinical research, there is no effective therapeutic intervention against the disease. Lipopolysaccharide (LPS), a component of the outer cell wall in Gram-negative bacteria (3), activates intracellular signaling pathways via Toll-like receptor (TLR) 4. CpG DNA, contained in microbial DNA sequences, is recognized by TLR9. LPS and CpG DNA act as pathogen-associated molecular patterns (PAMPs) to develop effects independently or synergistically and are potent triggers of inflammation, eventually causing systemic inflammatory response syndrome (SIRS) and sepsis (4–7). LPS and CpG DNA therefore play a key role in the initial cause of sepsis and may be targets for the treatment of sepsis. Treatment of sepsis has previously been attempted by targeting one of either of the two PAMPs, for example through the use of lipid A analogues or antibodies against endotoxin (8). The benefits of these drugs are still debatable as they may not be effective for all types of sepsis (9). It is therefore necessary to develop a more efficacious treatment for sepsis, and targeting LPS and CpG DNA simultaneously may be a suitable strategy.

Kukoamine B (KB, C_28_H_42_N_4_O_6_), a pure spermine alkaloid with polyamine backbone and dihydrocaffeic acid appendage, is extracted from a traditional Chinese herb, cortex Lycii, via a rapid screening technique based on a herb affinity assay. KB can immobilize LPS or CpG DNA separately on the reacting surfaces of a biosensor (10,11) and has been found to exhibit a high affinity with both LPS and CpG DNA (12,13); however, little is known regarding whether the *in vivo* administration of KB can effectively inhibit inflammation in septic mice. The aim of the present study was therefore to investigate the inhibitory effects of KB on the inflammatory response in the livers of LPS-induced septic mice.

## Materials and methods

### Ethics statement

The present study was approved by the Council on Animal Care and the Protection and Welfare of Animals at Jiangsu University (Zhenjiang, China). All experimental protocols followed the National Institutes of Health of China guidelines for the care and use of experimental animals.

### Reagents and materials

**Figure 1:**
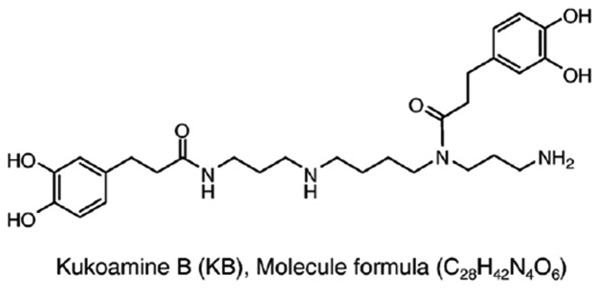
Chemical structure of kukoamine B.

KB was kindly provided by Dr Zhen Jiang (Third Military Medical University, Chongqing, China). The chemical structure of KB is shown in Fig. 1. LPS, from *Escherichia coli* 055:B5, was purchased from Sigma Chemicals (St. Louis, MO, USA). LPS was diluted in normal saline and was administered to the animals intraperitoneally. Interleukin-1β (IL-1β) and tumor necrosis factor α (TNF-α) ELISA kits were purchased from Joyee Biotechnics Co., Ltd. (Shanghai, China). The myeloperoxidase (MPO) assay kit was obtained from Nanjing Jiancheng Bioengineering Institute (Nanjing, China). Limulus amebocyte lysat (LAL) reagents were obtained from Xiamen Houshiji Co., Ltd. (Xiamen, China). All other chemicals were of reagent grade and obtained from Sigma Chemicals, unless otherwise stated.

### Animals and experimental sepsis

Institute of Cancer Research mice (body weight, 20±2 g; Experimental Animal Center of the Jiangsu University) were kept in the animal house in a temperature-controlled room and were allowed free access to normal animal diets and tap water. The mice were left for a four-day acclimation period prior to the beginning of the experiments. Thirty-two mice were randomly divided into three groups: Control (n=8), LPS (n=12) and LPS + KB (n=12). Mice in the LPS group were injected with LPS (10 mg/kg, intraperitoneally) (14). In the LPS + KB group, KB (20 μg/kg, intravenously) was administered 4 h after the LPS challenge for a further 4 h. Control animals received only a vehicle.

### Measurement of plasma LPS concentration

At 0, 2 and 4 h after KB treatment, the concentration of LPS in the plasma was determined using the LAL test. Briefly, each sample (100 μl) was added into 100 μl quantitative LAL reagents dissolved in LPS-free water and reacted at 37°C for 2 h. The gel clotting formation of the LAL products induced by the existence of non-neutralized LPS was measured through the kinetic turbidimetric assay, in which endodoxin triggers a cascade of enzymatic reactions to activate the clotting enzyme. The formation of the gel clot is proportional to the concentration of endotoxin in the sample. Aliquots (100 μl) of all samples, standards and negative controls were seeded into a 96-well plate (non-pyrogens) and incubated at 37°C in a BioTek ELx808 reader (BioTek Instruments, Inc., Winooski, VT, USA). for 10 min. Following incubation, ~100 μl quantitative LAL reagents, dissolved in LPS-free water, rotate up and down until the solution turned clear prior to use, was added to each well (Chinese Horsehoe Crab Reagent Manufacturery Co., Ltd., Xiamen, China). Following gentle vibration for 10 sec, the absorbance at 630 nm was measured and readings were repeated every 30 sec for 2 h. The results were calculated using Gen5™ data analysis software (BioTek Instruments, Inc.).

### Measurement of serum levels of transaminases

At 4 h after KB treatment, the mice were anesthetized with spontaneous inhalation of isoflurane-N_2_O (Abbott Laboratories, Missisauga, ON, Canada) in a 60% O_2_/40% N_2_ mixture. Blood samples were obtained by cardiac puncture of the left ventricle. The samples were stored in serum tubes (Capiject^®^; Terumo Medical Corporation, Somerset, NJ, USA) and immediately centrifuged at 6,500 × g for 5 min. The serum levels of alanine aminotransferase (ALT) and aspartate aminotransferase (AST) were measured using commercially available clinical assay kits according to the manufacturers’ instructions and determined by a serum autoanalyzer (AU2700; Olympus Corp., Tokyo, Japan). ALT catalyzes the transfer of the amino group from L-alanine to α-ketoglutarate resulting in the formation of pyruvate and L-glutamate. Lactate dehydrogenase catalyzes the reduction of pyruvate and the simultaneous oxidation of NADH to NAD decreasing the absorbance, directly proportional to ALT activity. AST catalyzes the transfer of an amino group between L-aspartate and 2-oxoglutarate. The oxalacetate formed in the first reaction then reacts with NADH in the presence of malate dehydrogenase (MDH) to form NAD. The resulting decrease in absorbance is directly proportional to AST activity.

For ALT, reagent 1 (R1) contained 100 mM Tris buffer, 0.18 mM NADH 15 mM α-ketoglutaric acid, 1,200 U/l LDH. R2 contained 240 mM L-alanine. For AST, R1 contained 100 mM Tris buffer, 12 mM α-ketoglutaric acid, 0.18 mM NADH, >2,000 U/l LDH and 1,2000 U/l MDH; R2 contained 240 mM L-aspartate. A 4:1 mixture of R1 reagent (100 mM Tris buffer, 0.18 mM NADH 15 mM α-ketoglutaric acid, 1,200 U/l LDH) and R2 reagent (240 mM L-alanine) was pipietted (1.0 ml) into appropriate tubes. Subsequently, 40 μl of the samples, calibrator and negative control to the reagent was mixed and incubated at 37°C for 1 min. The absorbance was measured using a spectrophotometer at 340 nm and the mixture was returned to 37°C. The readings were repeated every 1 min for a total of 9 min. The mean absorbance difference/minute (ΔA/min.) was calculated and expressed as activity U/l. This was calculated using the formula: Activity = ΔAu/min / ΔAc/min * Cc. ΔAu/min deontes the mean absorbance difference/minute of the sample, ΔAc/min denotes the mean absorbance difference/minute of the calibrator, Cc denotes the concentration of the calibrator. The commercially available clinical assay kits were from Sichuan Maker Biotechnology Co., Ltd (Sichuan, China).

### Histopathological examination

To characterize any histological alterations, the livers were harvested from the animals of the different groups 4 h after KB treatment and fixed in 4% formaldehyde solution. The tissue was dehydrated with graded alcohol and embedded in paraffin, and the sections were then stained with hematoxylin and eosin. The tissue morphological characteristics were examined using light microscopy.

### Preparation of tissue homogenates

Mice were sacrificed 4 h after KB treatment. The livers were immediately collected and stored at −80°C. Equal weights of liver tissue from the three groups were homogenized in ice-cold 0.9% NaCl to yield a 10% (w/v) homogenate. The homogenates were then cleared by centrifuging at 9,000 × g at 4°C. The supernatants were obtained and stored at −70°C.

### ELISA

The levels of TNF-α and IL-1β in the plasma and tissue homogenates were measured using ELISA kits in accordance with the manufacturer’s instructions (Joyee Biotecnics Co., Ltd., Shanghai, China.).

### MPO activity

To assess the neutrophil infiltration, the MPO activity in the tissue homogenates was determined by utilizing a commercially available kit in accordance with the manufacturer’s instruction (Nanjing Jiancheng Bioengineering Institute, Nanjing, China) (15). Aliquots (0.3 ml) were added to a 2.3-ml reaction mixture containing 50 mM potassium phosphate buffer, o-dianisidine and 20 mM H_2_O_2_ solution. One unit of enzyme activity (expressed as U/g tissue) was defined as the amount of MPO required to cause a change in absorbance measured at 460 nm for 3 min.

### Immunohistochemical examination

The paraffin-embedded liver tissue sections were subjected to immunohistochemical staining. Briefly, paraffin sections of 5 μm were prepared and mounted on SuperFrost™ Plus glass slides (Kaihong Healthcare Co., Ltd., Nanjing, China), and were then deparaffinized in xylene and rehydrated in a graded series of ethanol baths. Endogenous peroxidase activity in the deparaffinized sections was blocked through treatment with 3% H_2_O_2_. Following the termination of endogenous peroxidase activity, the nonspecific proteins were blocked with 3% solcoseryl for 30 min at room temperature. The sections were subsequently incubated with goat polyclonal immunoglobulin (Ig)G primary antibodies against intercellular adhesion molecule-1 (ICAM-1; sc-1511) and vascular cell adhesion molecule-1 (VCAM-1; sc-1504) (Santa Cruz Biotechnology, Inc., Santa Cruz, CA, USA) diluted 1:200 in Tris buffered saline containing 0.1% Tween-20 (TBST) at 4°C overnight. The following morning the secondary antibodies [rabbit anti-goat immunoglobulin (Ig) G conjugated with horseradish peroxidase], obtained from Maixin Biotech Co., Ltd. (Fuzhou, China) were used to bind with the primary antibodies. The location of the stained proteins was subsequently determined by reaction with 3′-diaminobenzidine tetrahydrochloride solution according to the manufacturer’s instructions (Sigma Chemicals) and examined by light microscopy.

### Western blot analysis

Total nucleic protein was extracted with a nuclear protein extraction buffer kit (Vazyme Biotech, Nanjing, China). Protein concentration was assayed using a bicinchoninic acid protein assay kit (Beyotime Institute of Biotechnology, Haimen, China). SDS-PAGE was performed on equivalent amounts of protein samples using precast 7% resolving/3% stacking Tris-HCl gels (Bio-Rad, Hercules, CA, USA). Following electrophoresis, the separated proteins were transferred to polyvinylidene fluoride membranes (Amersham Pharmacia Biotech, Inc., Piscataway, NJ, USA), prior to the membranes being blocked in 5% non-fat milk in TBST for 1 h at room temperature. The blocked membranes were incubated in rabbit polyclonal IgG primary antibodies specific for mouse nuclear factor-κ-gene binding (NF-κB)-p56 (sc-372 at a dilution of 1:1,000), in TBST overnight at 4°C. The membranes were then washed and probed with horseradish peroxidase-conjugated secondary antibody (Amersham Pharmacia Biotech, Inc.) for 1 h at room temperature. Chemiluminescence detection was performed with the Amersham enhanced chemiluminescence detection kit according to the manufacturer’s instructions (Amersham Pharmacia Biotech, Inc.). To ensure a similar quantity of protein in each sample, the membranes were ‘stripped off’, reprobed with β-actin, developed with horseradish peroxidase-conjugated secondary antibody and visualized using enhanced chemiluminescence. The specific bands were quantified by densitometry (Bio-Rad GS-710 densitometer; Bio-Rad Laboratories, Des Moines, IA, USA).

### Electrophoretic mobility shift assay (EMSA)

Whole-tissue (medial lobe of the liver) nuclear protein was extracted as described previously (16,17). For EMSA, 10 μg total nuclear protein was incubated with 1.0 pmol double-stranded γ-[^32^P]-adenosine triphosphate end-labeled oligonucleotides containing consensus-binding sequences for NF-κB (sense strand 5′-AGGGACTTCCGCTGGGGACTTTCC-3′) in a binding buffer (10 mM HEPES, pH 7.9, 80 mM NaCl, 3 mM MgCl_2_, 0.1 mM EDTA, 1 mM dithiothreitol, 1 mM phenylmethylsulfonyl fluoride and 10% glycerol). The samples were incubated for 30 min at room temperature and then run through a 4% non-denaturing polyacrylamide gel (0.5X Tris-borate-EDTA buffer) at 280 V for 1 h. Once dry, the gel was exposed to X-ray film (Kodak, Rochester, NY, USA) for 4–6 h in cassettes at −80°C. Signal detection and quantification was performed by computer-assisted densitometry.

### Statistical analysis

All values are expressed as the mean ± standard deviation and were analyzed using one-way analysis of variance, with a post hoc Dunnett’s t-test, and a two-tailed Student’s t-test. Differences between groups were considered to be statistical significant at P<0.05.

## Results

### Effect of KB on LPS concentration in the plasma of LPS-induced septic mice

**Figure 2:**
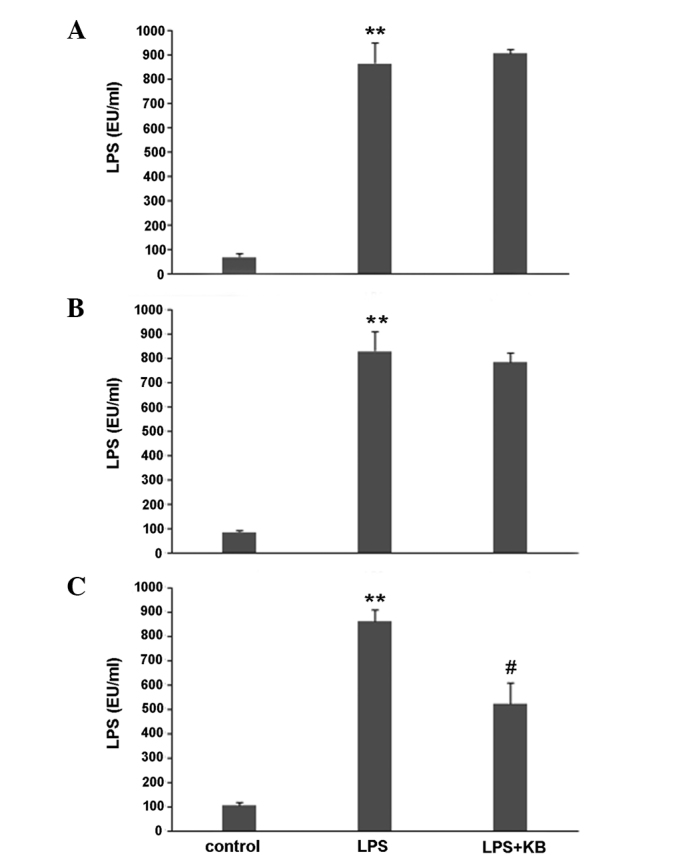
Effect of KB on LPS concentration in the plasma of LPS-induced septic mice. Mice were challenged with LPS for 4 h and then treated with KB for 0, 2 and 4 h. The concentration of LPS was determined using a kinetic turbidimetric assay. Mice challenged with LPS had a significantly higher LPS concentration than the control group. (A) In mice challenged with LPS followed by KB administration for 0 h, no significant reduction in LPS was observed. (B) LPS concentration decreased following KB treatment for 2 h. (C) The decrease in LPS concentration was significant following KB treatment for 4 h. Results are presented as the mean ± standard deviation. ^**^P<0.01 compared with control mice. ^#^P<0.05 compared with LPS mice. KB, kukoamine B; LPS, lipopolysaccharide; EU, endotoxin units.

After a 4-h challenge with LPS, mice were treated with KB for 0, 2 and 4 h. The LPS concentrations in the plasma were determined. As shown in Fig. 2, mice challenged with LPS had a significantly higher LPS concentration compared with the control group. The LPS concentration declined following KB treatment for 2 h (Fig. 2B). The decrease in LPS concentration was significant following KB treatment for 4 h (Fig. 2C).

### Histology

**Figure 3:**
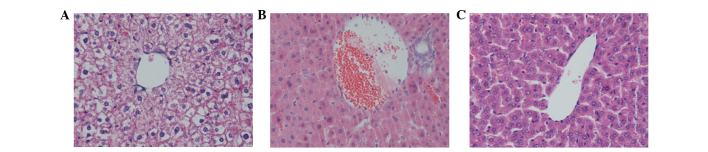
Effect of KB on liver injury in LPS-induced septic mice (hematoxylin and eosin staining). Mice were challenged with LPS for 4 h and then treated with KB for 4 h. Histological analysis was performed. Compared with (A) the control group, (B) LPS-challenge induced hepatocyte arrangement irregularities and central vein congestion, as well as the infiltration of inflammatory cells into the tissue. (C) Administration of KB significantly decreased the granulocyte infiltration and inflammatory response. The figure is representative of at least three experiments performed on different days. (Magnification, ×400). KB, kukoamine B; LPS, lipopolysaccharide.

Histological analysis showed that the livers from the control mice exhibited the normal architecture, while LPS challenge induced irregularity in hepatocyte arrangement and central vein congestion, as well as the infiltration of inflammatory cells into the tissue. Administration of KB significantly decreased the granulocyte infiltration and inflammatory response (Fig. 3).

### Effect of KB on serum aminotransferase levels and liver MPO activity in LPS-induced septic mice

**Figure 4:**
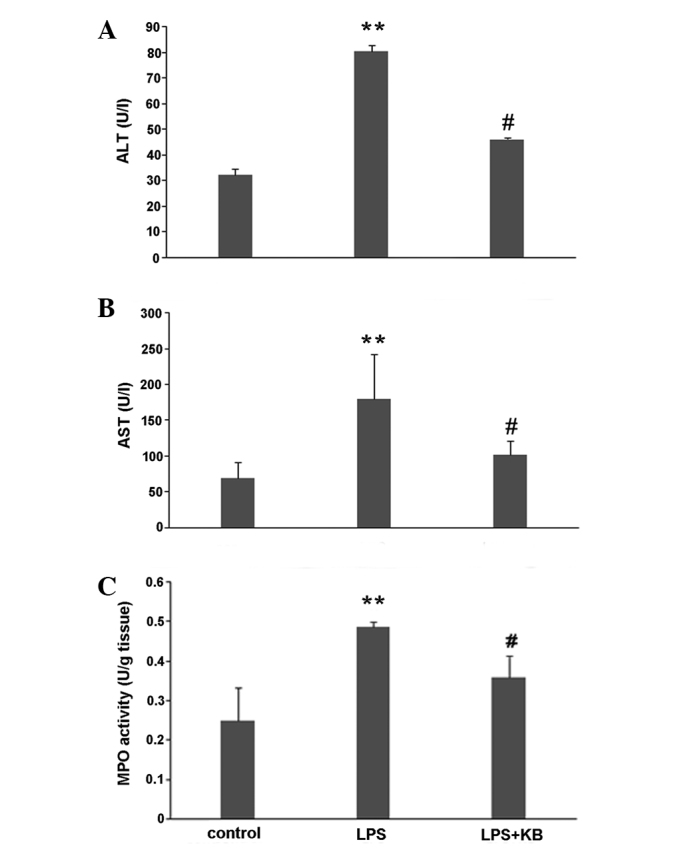
Effect of KB on serum aminotransferase levels and liver MPO activity in LPS-induced septic mice. Mice were challenged with LPS for 4 h and then treated with KB for 4 h. Serum aminotransferase levels and MPO activity in the liver were assessed 4 h after KB treatment. (A and B) Levels of (A) ALT and (B) AST in the LPS-induced septic mice were markedly increased. Following the administration of KB, this elevation was significantly attenuated. (C) MPO activity in the livers obtained from LPS-induced septic mice was markedly increased compared with that in the control animals, whereas the activity was significantly decreased by treatment with KB. Results are presented as the mean ± standard deviation. ^**^P<0.01 compared with control mice. ^#^P<0.05 compared with LPS mice. KB, kukoamine B; LPS, lipopolysaccharide; ALT, alanine aminotransferase; AST, aspartate aminotransferase; MPO, myeloperoxidase.

Hepatocyte injury was evaluated by determining the serum concentrations of ALT and AST. As shown in Fig. 4A and B, the levels of ALT and AST in LPS-induced septic mice were found to be markedly increased. Following the administration of KB, this elevation was significantly attenuated (compared with the LPS group, P<0.05). To determine whether the LPS-induced increase in polymorphonuclear leukocyte (PMN) accumulation in the liver was effectively prevented by KB, the activity of MPO, an enzyme in the azurophilic granules of neutrophils, was assessed. The mean MPO levels are shown in Fig. 4C. The MPO activity in the livers obtained from LPS-induced septic mice was markedly increased compared with that in the control animals (P<0.01), whereas the activity was significantly decreased by treatment with KB (P<0.05).

### Effect of KB on cytokine expression in LPS-induced septic mice

**Figure 5:**
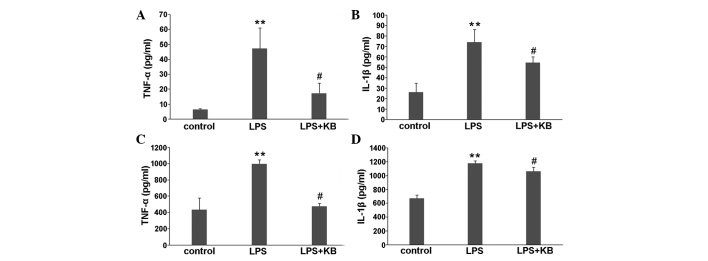
Effect of KB on cytokine expression in LPS-induced septic mice. Mice were challenged with LPS for 4 h and then treated with KB for 4 h. TNF-α and IL-1β levels in the plasma and tissue homogenates were assayed 4 h after KB treatment using ELISA kits. The levels of TNF-α and IL-1β in the (A and B) plasma and (C and D) tissue homogenates were markedly elevated following LPS challenge compared with those in the control group. Following the administration of KB, the increases in the cytokine levels were significantly attenuated. Results are presented as the mean ± standard deviation. ^**^P<0.01 compared with control mice. ^#^P<0.05 compared with LPS mice. KB, kukoamine B; LPS, lipopolysaccharide; TNF-α, tumor necrosis factor α; IL-1β, interleukin-1β.

To evaluate the inflammatory response, levels of TNF-α and IL-1β in the plasma and liver homogenates were detected. The expression of TNF-α and IL-1β in the plasma of LPS-induced septic mice was markedly elevated compared with that in the plasma of the control mice. Following the administration of KB for 4 h, the increases in TNF-α and IL-1β expression were significantly reduced (Fig. 5A and B). Similar results for TNF-α and IL-1β were found in the liver homogenates (Fig. 5C and D).

### Effect of KB on ICAM-1 and VCAM-1 expression in the livers of LPS-induced septic mice

**Figure 6:**
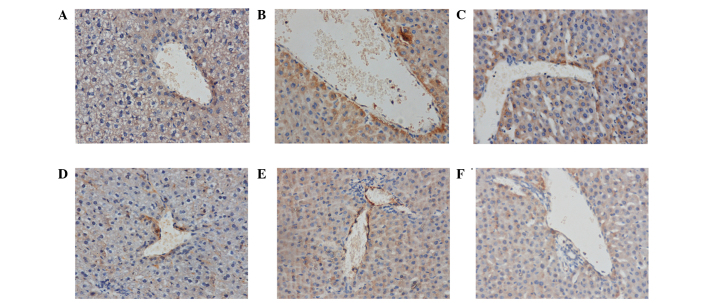
Effect of KB on ICAM-1 and VCAM-1 expression in the livers of LPS-induced septic mice. Mice were challenged with LPS for 4 h and then treated with KB for 4 h. The expression of the cellular adhesion molecules ICAM-1 and VCAM-1 was determined by immunohistochemical stains 4 h after KB treatment. Following LPS stimulation, the expression of ICAM-1 and VCAM-1 in the liver tissue was significantly increased compared with that in the control; however, with the *in vivo* administration of KB, the expression of (A-C) ICAM-1 and (D-F) VCAM-1 decreased significantly. (A and D) control group; (B and E) LPS group; (C and F) LPS + KB group. (Magnification, ×400). KB, kukoamine B; LPS, lipopolysaccharide; ICAM-1, intercellular adhesion molecule-1; VCAM-1, vascular cell adhesion molecule-1.

Following LPS stimulation, the expression of ICAM-1 and VCAM-1 in the liver tissue was significantly increased compared with that in the control. With the *in vivo* administration of KB, the expression of ICAM-1 and VCAM-1 significantly decreased (Fig. 6).

### Effect of KB on NF-κB activity in LPS-induced septic mice

**Figure 7:**
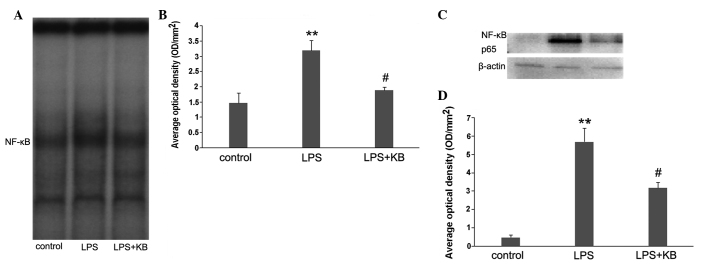
Effect of KB on NF-κB activity in LPS-induced septic mice. Mice were challenged with LPS for 4 h and then treated with KB for 4 h. (A and B) The binding activity of nuclear protein to the radio-labeled consensus binding sequences of NF-κB was assessed by electrophoretic mobility shift assay. The NF-κB activation in the livers of LPS-challenged mice was markedly increased, and this activity was inhibited by *in vivo* administration of KB. (C and D) Equal quantities of nuclear protein were used for western blot analysis with the indicated antibodies. Levels of NF-κB-p65 were markedly elevated in the nuclear protein of LPS-challenged mice, while KB treatment attenuated this elevation. Experiments were performed three times. (A and C) Representative images; (B and D) average optical densities. Results are presented as the mean ± standard deviation. ^**^P<0.01 compared with control mice. ^#^P<0.05 compared with LPS mice. KB, kukoamine B; LPS, lipopolysaccharide; NF-κB, nuclear factor κ-gene binding.

The binding activity of nuclear protein to the radio-labeled consensus binding sequences of NF-κB was assessed using EMSA. Following LPS challenge, the NF-κB activation in the liver was markedly increased, and this activity was inhibited by the *in vivo* administration of KB (Fig. 7A and B). The nuclear translocation of the p65 subunit of NF-κB was subsequently investigated using western blot analysis to conform the effect of KB on NF-κB-p65 translocation from the cytosol to the nucleus. The data indicated that levels of NF-κB-p65 were markedly elevated in the nuclear protein of LPS-challenged mice; KB treatment attenuated this elevation (Fig. 7C and D).

## Discussion

KB, an active alkaloid compound isolated from the traditional Chinese herb cortex Lycii, is considered to be a novel and promising candidate for the treatment of sepsis (12). KB is firstly characterized as a selective dual inhibitor of LPS and CpG DNA (13). KB possesses anti-inflammatory activity, as demonstrated by its ability to inhibit the inflammatory response in mouse macrophages, rescue mice from heat-killed *E. coli-*induced sepsis and prevent the upregulation of TLR4 and TLR9 expression (12,13). These data provide evidence that, as a potential LPS-neutralizer, KB affects the signal transduction pathway activation stimulated by LPS; however, to the best of our knowledge, no studies have assessed the anti-LPS ability of KB following its administration in LPS-challenged mice. The aim of the present study was therefore to focus on the anti-inflammatory effects of KB in the livers of LPS-challenged mice and to explore a potential mechanism. In the study it was found that the plasma LPS concentration reached a high level in LPS-challenged mice. As expected, the LPS level was significantly decreased in the LPS-challenged mice treated with KB for 4 h. These data are consistent with those in previously published studies (12).

Exerting important roles in metabolism, homeostasis and host defense mechanisms, the liver has been investigated extensively and is believed to be a major organ responsible for SIRS or sepsis (18). Liver injury in LPS-challenged mice is characterized by neutrophil infiltration in the liver parenchyma, irregularity in the arrangement of numerous hepatocytes and central and portal vein congestion. In the present study, it was observed that these histological changes were slight in the LPS + KB group. In parallel, the elevated levels of the hepatic enzymes AST and ALT were effectively reduced in the LPS-challenged mice treated with KB, indicating that KB administration plays a key role in the anti-inflammatory response and the protection of organ functions.

The excessive production of certain cytokines, such as TNF-α and IL-1β, released by macrophages and other mononuclear cells in response to LPS is understood to be one of the earliest events in hepatic inflammation. This cytokine production triggers a cascade of other cytokines that act in coordination to cause the death of hepatocytes and the recruitment of inflammatory cells (19–21). To investigate whether suppression of the LPS-induced liver inflammatory response by KB was due to a downregulation in the expression of systemic and local proinflammatory cytokines, the expression of TNF-α and IL-1β was measured in plasma and liver tissue in the experimental mice of the present study. *In vivo* application of KB in the LPS-challenged mice markedly decreased the production of TNF-α and IL-1β in the plasma and liver tissue. These data indicated that the activation and release of proinflammatory mediators in systemic and local tissue could be, at least partly, inhibited by KB, and that this process could be associated with the decreased LPS level following neutralization by KB.

In addition to the expression of proinflammatory cytokines, neutrophil sequestration was investigated in the present study. Neutrophil-mediated parenchymal cell damage in the liver is initiated by the accumulation of neutrophils in the hepatic tissue (22). MPO is an enzyme that can predominantly be found in the azurophilic granules of PMNs. The measurement of tissue MPO activity is frequently utilized to reflect the PMN accumulation in damaged tissues, since MPO activity correlates significantly with the number of PMNs determined histochemically in tissues (23,24). The present results showed that MPO activity in the liver was markedly enhanced following LPS stimulation. *In vivo* administration of KB led to a significant decrease in MPO activity, subsequently preventing PMN chemotaxis and infiltration in the liver, and decreasing the production of oxidants and tissue oxidative injury.

ICAM-1 and VCAM-1 are two members of the Ig-like supergene family of adhesion molecules, which are responsible for mediating the tight adhesion of PMNs to endothelial cells and assisting leukocyte transmigration (25–27). In the present study, the expression of ICAM-1 and VCAM-1 was detected in the livers of experimental mice. The data showed that KB inhibited the increases in ICAM-1 and VCAM-1 expression in the livers of LPS-induced septic mice. These results were in accord with the changes in MPO activity, and strongly indicated that KB is associated with the inhibition of leukocyte sequestration and adhesion, and may consequently effectively decrease the inflammatory response in livers stimulated by LPS.

The binding of LPS to host cells induces a receptor-mediated (TLR4) signaling cascade that leads to the activation of NF-κB. Following activation, NF-κB translocates to the nucleus and causes rapid gene induction, resulting in the expression of inflammatory mediators, including cytokines, chemokines and adhesion molecules (28–30). To further explore the underlying mechanism by which KB achieves its beneficial effects, NF-κB translocation and activity, which play an important role in the pathogenesis of liver injury, were investigated. The present study demonstrated that NF-κB translocation was strongly increased, and accompanied by enhanced NF-κB activity, in the liver tissue of LPS-induced septic mice. Notably, KB effectively attenuated the nuclear translocation and activation of NF-κB, indicating that KB has a pivotal role in the inhibition of NF-κB via its ability to bind to LPS, subsequently leading to the alleviation of leukocyte infiltration and the LPS-induced proinflammatory response.

In combination, the results of the present study have demonstrated that KB inhibits inflammation in septic mice by its unique property of combining with LPS, leading to a reduction in the concentration of plasma LPS. Decreases in the plasma LPS levels were accompanied by reductions in the expression of ICAM-1 and VCAM-1 and leukocyte sequestration in the livers of LPS-induced septic mice. In parallel, KB was shown to exert its protective effects against the inflammatory response by interfering with NF-κB activation, and, therefore, suppressing the pro-adhesive phenotype of the endothelial cells. Further studies are required in extension of the present observations in order to investigate the detailed mechanism(s) regarding KB in the treatment of sepsis.
